# Anti-Rheumatic Effect of Antisense Oligonucleotide Cytos-11 Targeting TNF-α Expression

**DOI:** 10.3390/ijms22031022

**Published:** 2021-01-20

**Authors:** Tatyana P. Makalish, Ilya O. Golovkin, Volodymyr V. Oberemok, Kateryna V. Laikova, Zenure Z. Temirova, Olesya A. Serdyukova, Ilya A. Novikov, Roman A. Rosovskyi, Andrey I. Gordienko, Evgeniya Yu. Zyablitskaya, Elvina A. Gafarova, Kseniya A. Yurchenko, Iryna I. Fomochkina, Anatoly V. Kubyshkin

**Affiliations:** 1 Medical Academy Named after S.I. Georgievsky, V.I. Vernadsky Crimean Federal University, Lenin Boulevard 5/7, 295051 Simferopol, Russia; makalisht@mail.ru (T.P.M.); wwwzzznnn333@gmail.com (Z.Z.T.); uu4jey@mail.ru (A.I.G.); evgu79@mail.ru (E.Y.Z.); gafarova.elvina@inbox.ru (E.A.G.); yurchenkokseniya28@gmail.com (K.A.Y.); fomochkina_i@mail.ru (I.I.F.); kubyshkin_av@mail.ru (A.V.K.); 2 Taurida Academy, V.I. Vernadsky Crimean Federal University, Vernadsky Av. 4, 295007 Simferopol, Russia; botan_icus@mail.ru (K.V.L.); aliss.serdyuckova@yandex.ru (O.A.S.); i.nowikow2012@mail.ru (I.A.N.); roman.rosovsky@yahoo.com (R.A.R.); 3 Nikita Botanical Gardens—National Scientific Centre Russian Academy of Sciences, 298648, Simferopol, Russia; 4 Research Institute of Agriculture of Crimea, 295005 Simferopol, Russia

**Keywords:** rheumatoid arthritis, antisense technologies, TNF-α, Humira^®^, phosphorothioate oligonucleotides, inflammation

## Abstract

The urgency of the search for inexpensive and effective drugs with localized action for the treatment of rheumatoid arthritis continues unabated. In this study, for the first time we investigated the Cytos-11 antisense oligonucleotide suppression of TNF-α gene expression in a rat model of rheumatoid arthritis induced by complete Freund’s adjuvant. Cytos-11 has been shown to effectively reduce peripheral blood concentrations of TNF-α, reduce joint inflammation, and reduce pannus development. The results achieved following treatment with the antisense oligonucleotide Cytos-11 were similar to those of adalimumab (Humira^®^); they also compared favorably with those results, which provides evidence of the promise of drugs based on antisense technologies in the treatment of this disease.

## 1. Introduction

Currently, rheumatoid arthritis is one of the most common chronic joint inflammation conditions worldwide, with an incidence of 0.5–2% among all ethnic groups [1,2,3]. No specific cause for the development of this disease has been established, but it is believed to be a combination of genetic predisposition and an interaction with environmental factors [4,5]. The disease process is accompanied by a decrease in the quality of life, beginning with pain and stiffness in the joints, sleep disturbances and depression, increased fatigue, and decreased labor productivity [6,7,8,9]. In the absence of treatment, it ends in early disability and complete immobilization of the affected joints [2]. The economic burden of rheumatoid arthritis is significant both for people with the disease and for health care facilities and is associated with high medical costs and a loss of performance among patients [6,10]. It should also be noted that the juvenile form of the disease is a serious problem in children. This disease, which ranks first among inflammatory diseases of the joints, is characterized by the involvement of vital organs in some children, complications and side effects of basic therapy, and a decreased quality of life [11,12].

Today, several groups of drugs are used to treat rheumatoid arthritis. Non-steroidal anti-inflammatory drugs quickly and effectively relieve the symptoms of the disease, such as pain and swelling of the joints, but have a limited effect on the destructive processes in the joints. Steroid drugs effectively suppress both destructive processes and the inflammatory response [13,14] but have many unwanted side effects, such as growth retardation, hyperglycemia, and allergic reactions, among others [15,16]. A revolution in the treatment of rheumatoid arthritis occurred when drugs aimed at inhibiting pro-inflammatory cytokines were introduced, especially those targeting tumor necrosis factor alpha (TNF-α), which is involved in most of the studied inflammatory processes. Based on determination of the amount of TNF-α in the peripheral blood, it is possible to assess the severity of the course of rheumatoid arthritis [17].

Adalimumab (Humira^®^) is currently the gold standard for treatment. Compared to its predecessor analogs, such as infliximab or etanercept, it is less immunogenic and less likely to cause allergic reactions [13]. Within an hour of taking infliximab, it is not uncommon for patients to experience headache, fever, hypotension, or vomiting. In about 20% of patients, depending on the dose, antibodies against the drug are produced; in these patients, the frequency of infusion reactions reaches up to 60% [18,19]. Etanercept causes fewer reactions after drug administration, the most frequent of which are local reactions at the injection site [20]. Since it is a chimeric protein created from the TNF-α receptor and human IgG1 [21], antibodies against it are produced less often, unlike infliximab, which is the combination of an Fc-fragment of human IgG1 and a mouse Fab-region [22]. Adalimumab, on the other hand, is a human monoclonal antibody against TNF-α, identical to IgG1, which binds in peripheral blood or on cell membranes with high efficiency and low immunogenicity [23]. The use of adalimumab in combination with drugs from other groups significantly increases the effectiveness of treatment and avoids irreversible damage to the joints [24,25]. However, adalimumab is rather expensive and, like infliximab and etanercept, has side effects associated with its systemic action, which limits its use. The most common adverse event associated with use of adalimumab is an increased risk of developing serious infectious diseases such as pneumonia, tuberculosis, and other pulmonary diseases [26,27,28]; according to some data, the frequency of developing serious infections may be increased by 1.5- to 2-fold [28,29].

Since adalimumab and similar drugs have immunosuppressive potential, attention should be focused of the need for more detailed research on how these drugs affect the risk of developing infections and the severity of those infections, including viral disease. It is now known that taking TNF-α inhibitors can affect the rate of reactivation of hepatitis B virus [30] or herpes virus [31]. However, although few studies have been done on viral respiratory infections, one study reported that patients taking TNF-α inhibitors were about 10% more likely to develop influenza-like illnesses [32]. Thus, the quest to find safe and effective drugs for the treatment of rheumatoid arthritis, with an efficacy similar to that of adalimumab, but a lower frequency of adverse reactions and a more localized effect, as well as a greater market availability, remains relevant.

In our opinion, the highest potential lies in drugs developed using antisense oligonucleotides (ASOs). Antisense technologies are based on the specific inhibition of unwanted gene expression by blocking or destroying mRNA activity [33,34]. They have several advantages over traditional drug therapies, such as the rapid and relatively affordable production of targeted oligonucleotides. In addition, the hydrogen bonds between the oligonucleotide and the complementary target mRNA are stronger than the bonds between different proteins; therefore, inhibition of gene expression leads to a longer lasting clinical effect [35]. As of 2020, more than 50 drugs based on antisense technologies are undergoing clinical trials, some of which are in Phase 2 and Phase 3 [33]. Currently, several drugs approved for treatment have been developed based on antisense technologies [36,37]; among these, nusinersen has shown very high efficacy in the treatment of spinal muscular atrophy without causing serious side effects [38]. The low frequency of side effects is associated with the structure of ASO-based drugs. Compared to typical biological drugs such as antibodies, ASOs are relatively small and have fewer potential epitopes; in addition, nucleic acids are considered relatively non-immunogenic, and a properly selected ASO sequence will affect only the target region, making its possible effects easy to predict [33,39]. Thus, the research and production of drugs based on antisense technologies is a promising area in medicine, with a real possibility of creating affordable drugs with high efficacy and low side effects.

Previous studies have been conducted on the effects of ASOs aimed at various targets to suppress the inflammation of the joints caused by rheumatoid arthritis. When ASOs aimed at a highly sensitive C-reactive protein (hs-CRP) were studied, they were found to have selectively reduced hs-CRP levels with a frequency of adverse reactions equal to that of the placebo group, but its utility as therapy in rheumatoid arthritis (RA) remains unclear [40]. In another study, the effect of an ASO aimed at proliferating cell nuclear antigen (PCNA) was studied, which was shown to be effective in suppressing the proliferation of synovitis in a cell model [41]. It is worth noting that one company developing drugs based on antisense technologies tested an ASO aimed at suppressing TNF-α expression, similar to our study presented in this article; however, in that study, a 20-mer methoxyethyl-modified ASO was used, whereas our 11-mer-modified ASO was without methoxyethyl protection. Their study showed that the ASO had an efficacy comparable to that of monoclonal antibodies against TNF-α and was well tolerated when tested in mice and in the first phase of clinical trials, but they stopped further research in 2005 in favor of other projects [42,43].

Reducing TNF-α activity has proven efficacy in the treatment of rheumatoid arthritis, and as antisense technologies are now an actively developing industry, the development of an ASO aimed at inhibiting TNF-α is both promising and timely. In our study, we synthesized an 11-mer phosphorothioate ASO aimed at inhibiting TNF-α and compared its effects on the dynamics of the course of rheumatoid arthritis in rats to the effects of adalimumab.

## 2. Results

**Figure 1:**
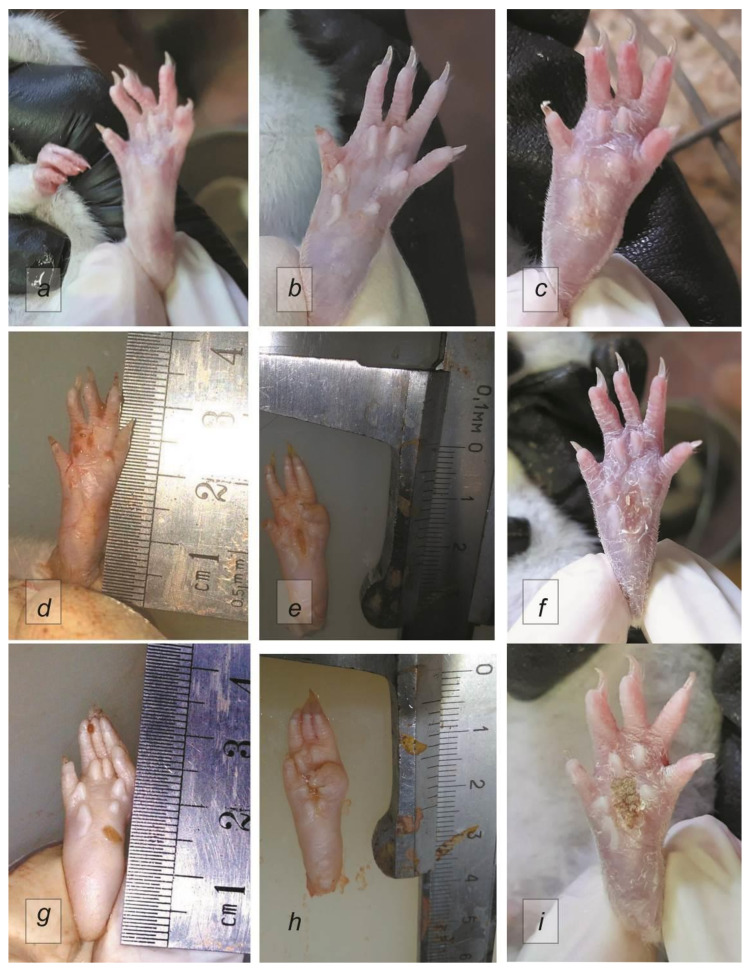
Left hind paws of rats. (**a**) Sham; (**b**,**c**) control after 7 and 21 days, respectively; (**d**–**f**) 7, 14, and 21 days after treatment with Cytos-11, respectively; (**g**–**i**) 7, 14, and 21 days after treatment with Humira, respectively.

According to the results of organometry carried out at the beginning of the experiment, there were no significant differences in the lengths of the thigh, lower leg, or foot among the different groups. Over the course of the experiment, there was a trend towards an increase in the vertical and transverse proportions of the foot in animals from the control group, while the use of Humira^®^ or Cytos-11 had a positive effect on reducing joint swelling. There was also a more pronounced swelling of the joints in the Humira group. There were no significant differences between the effects of the different drugs, not were there differences between the treatment groups and the Sham group (Figure 1).

### 2.1. Morphological Examination

At the beginning of the experiment, during morphological examination of the metatarsophalangeal joints, it was found that their condition corresponded to arthritis of 1–2 degrees of severity. Hyperplasia of synovitis and the development of the pannus were observed, containing, in addition to fibroblasts, lymphoid cells and macrophages. In the joint space of the individual joints, lymphoid infiltration was present, and the articular cartilage near the pannus was significantly thinned and had a fringed edge (Table 1).

**Figure 2:**
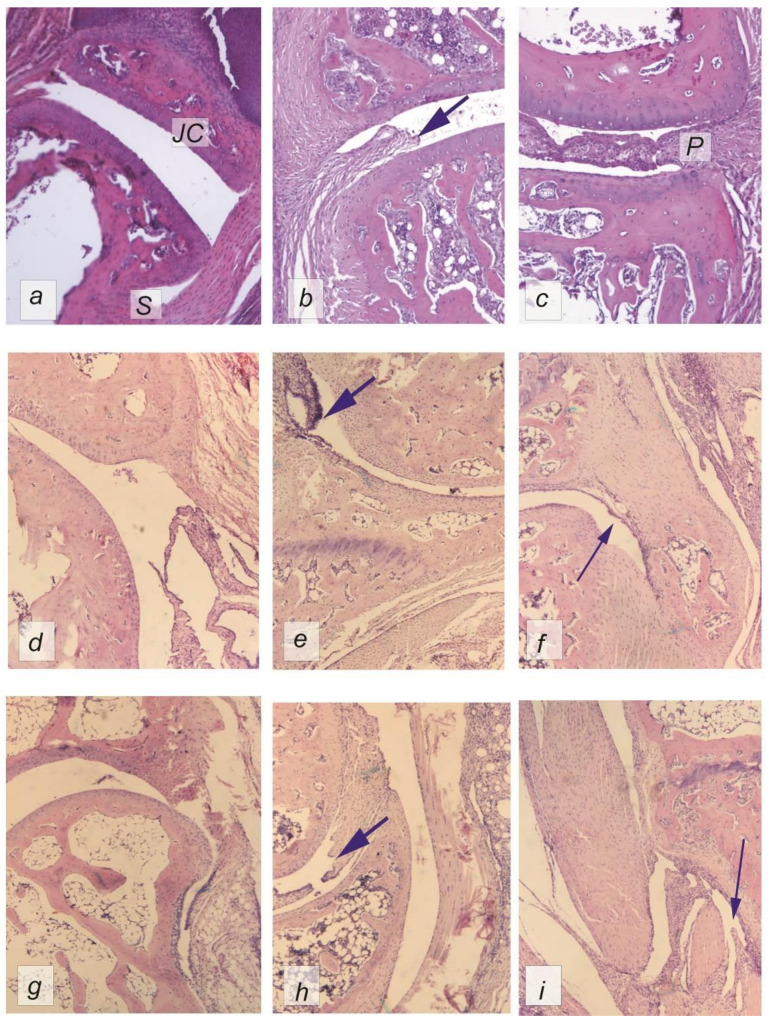
Hematoxylin–eosin-stained rat metatarsophalangeal joints from different periods during the experiment (used 5× lens); (**a**) sham; (**b**,**c**) control after 7 and 21 days, respectively; (**d**–**f**) 7, 14, and 21 days after treatment with Cytos-11, respectively; (**g**–**i**) 7, 14, and 21 days after treatment with Humira, respectively. Thick arrows indicate destructive changes in the joints and synovial hyperplasia. Thin arrows show the restoration of the normal structure of the synovium. P—pannus, S—synovial membrane, JC—joint cartilage.

In the group without treatment, we observed that the pannus developed faster than in the treatment groups during the experiment, the condition of the articular cartilage had worsened, and in some cases, bone tissue erosion had occurred. By Day 21, most of the animals in the control group had grade 3 arthritis (Figure 2b,c).

In the treatment groups on Day 7, an increase in the level of leukocyte infiltration in the pannus was observed (*p* < 0.05). We also observed synovial hyperplasia (*p* < 0.05) accompanied by the growing pannus (*p* < 0.05) (Figure 2d,g). However, the state of the cartilage tissue did not differ from that of the sham group, and there was no damage to the bone tissues, except for mild erosion of the tibia in the Humira group (*p* < 0.05).

By Day 14, pannus leukocyte infiltration remained (*p* < 0.05) in the control and Humira groups. The condition of the bone tissue had deteriorated in the Humira and Cytos-11 groups (*p* < 0.05), accompanied by synovial hyperplasia (*p* < 0.05), and an increase in the severity of the pannus (*p* < 0.05) (Figure 2e,h). Observations of the thickness of the articular cartilage on Day 14 revealed that it did not differ significantly among the groups, apart from the control group.

At the end of the experiment, on Day 21, there were no significant differences from the healthy animals in the treatment groups, apart from synovial hyperplasia in the Humira group (*p* < 0.05) (Figure 2f,i).

Multivariate analysis showed that the method of treatment had the strongest influence on the joint condition. This effect was most pronounced on indicators such as infiltration (F = 10.35; *p* < 0.05), the degree of pannus development (F = 10.97; *p* < 0.05), and synoviocyte hyperplasia (F = 10.79; *p* < 0.05).

In the control group, the joint cavity was occupied by the pannus, which had a border of hyperplastic synovitis with a tendency to increase over the course of the experiment; the arrow indicates bone erosion (Figure 2c).

### 2.2. Immunohistochemical Analysis

**Figure 3:**
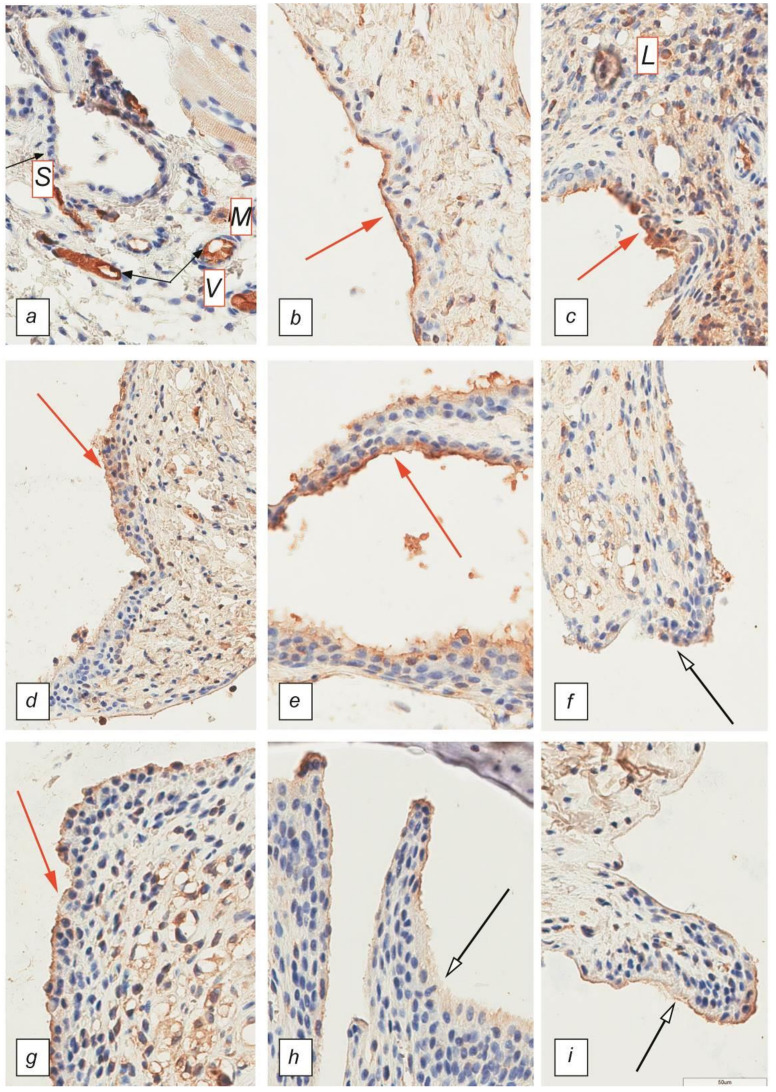
Synovial membrane of the metatarsophalangeal joints. Immunohistochemical staining with antibodies against TNF-α. Magnification 40×. (**a**) Sham; (**b**,**c**) control after 7 and 21 days; (**d**–**f**) 7, 14, and 21 days after treatment with Cytos-11; (**g**–**i**) 7, 14, and 21 days after treatment with Humira. L—lymphocytes, M—macrophages, S—synoviocytes, V—vascular endothelium. Red arrows indicate high TNF-α expression in untreated groups as well as early treatment with Cytos-11 and Humira. White arrows indicate a decrease in TNF-α expression.

Immunohistochemical staining of tissues for the presence of TNF-α revealed that in the sham group, synoviocytes did not express this cytokine, while in the surrounding tissues, single cells of the myeloid series, mainly macrophages, showed cytoplasmic staining of moderate intensity (Figure 3a). In the control group, there was an increase in the expression of TNF-α by cells of the lymphoid series in the tissues surrounding the joint during the experiment from 2 units on Day 7 to 3 units on Day 21; in addition, TNF-α is synthesized by synoviocytes with the progression of the disease, the staining intensity of which was estimated at an average of 2.5 units by the last day of the experiment (Figure 3b,c). Administration of the drug directly into the region of the joint had a positive effect on the joint, which was reflected by reduced expression of TNF-α synoviocytes and lymphocytes after 14 days of the experiment to 1 unit in the Cytos-11 group and an average of 0.5 units in the Humira group. After 21 days, there were no significant differences between the treatment groups and the sham group (Figure 3d–f).

In the sham group there was no positive staining of synoviocytes. Both single macrophages and vascular endothelium demonstrated moderate cytoplasmic color. In the control group, hyperplastic synoviocytes were expressed with a bright staining of the cytoplasm. Staining was also present in lymphocytes (Figure 3).

### 2.3. Linked Immunosorbent Assay

**Figure 4:**
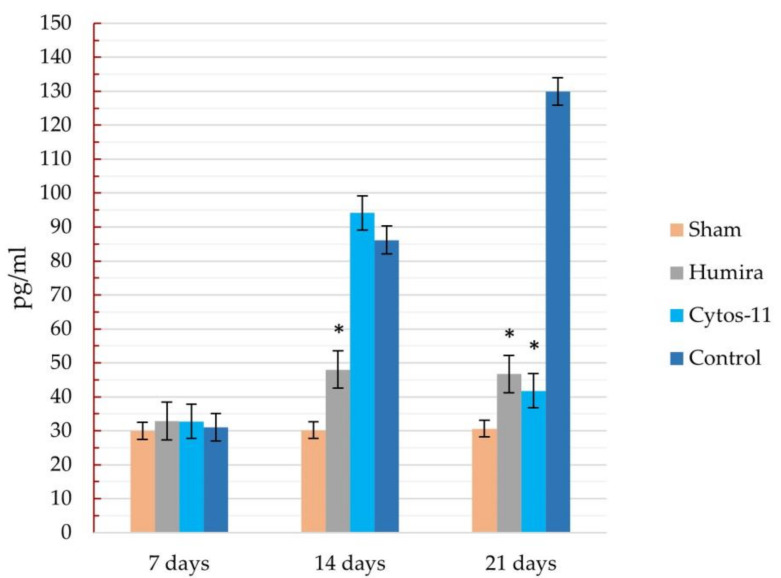
Dynamics of changes in the concentration of TNF-α in peripheral blood in different groups of the experiment. * Significant differences compared to the control group. I—error of the mean.

The concentration of TNF-α in the peripheral blood correlated with the results of immunohistochemical analysis and corresponded to the dynamics of the inflammatory process. At the beginning of the experiment, before dividing the rats into treatment groups, the mean concentration of TNF-α in the sham group was 30.3 pg/mL, and in the group with RA 31.6 pg/mL. On the 7th day of the experiment, there were no differences in the concentration of TNF-α among the groups, and the concentration of TNF-α was approximately 31 ± 2.1 pg/mL for all groups (Figure 4). On the 14th day, an increase in the concentrations of TNF-α was observed in animals with RA: 48.01 ± 11.73 pg/mL for the Humira group, 94.12 ± 9.69 pg/mL for the Cytos-11 group, and 86.15 ± 5.9 pg/mL for the control group. At the same time, there was an almost significant difference in indicators between the Humira group and the control group (*p* = 0.06). On the 21st day, the concentration of TNF-α had decreased in the treatment groups: 46.68 ± 7.67 pg/mL for the Humira group and 41.77 ± 7.8 pg/mL for the Cytos-11 group. This was significantly different from the group without treatment (*p* < 0.05), in which the concentration continued to increase to 129.8 pg/mL (±8.34).

## 3. Discussion

The great advantage of antisense strategies is their specificity. This makes the use of antisense technologies attractive in clinical practice. The ability of ASOs to selectively block the expression of target genes means that studying the pathogenesis of diseases and their underlying molecular pathways opens up many targets for therapeutic intervention using antisense technologies. This is especially desirable because in addition to causing selective effects on target genes, ASOs do not induce immunological factors [44]. ASOs such as Cytos-11 are a valuable addition to biologic and standard antirheumatic drugs, particularly adalimumab, making use of ASO technology, a promising method in the development of treatment for rheumatoid arthritis.

In rat models of RA (induced by Freund’s complete adjuvant), Cytos-11 was shown to reduce signs of joint inflammation. There was a decrease in edema around the affected joints, a decrease in morphological signs of the disease, and a decrease in TNF-α expression on cell membranes and in the peripheral blood. Comparison with adalimumab showed that Cytos-11 has a similar efficacy and that both drugs showed a major effect after 14 days of treatment. However, while organometric indices and morphological examination showed similar results on the same day, there were differences in immunohistochemical analysis and the concentrations of TNF-α. While Cytos-11 showed more significant results on the 21st day of treatment, it also had higher concentrations of TNF-α on the 14th day. Adalimumab selectively binds to TNF-α and neutralizes its biological functions; it is probable that the mechanism of action of this drug explains the low degree of immunohistochemical staining and levels of TNF-α in peripheral blood in our experiment, since the antigen is bound, making it inaccessible for detection when stained with antibodies during enzyme immunoassay analysis. Additionally, due to some inertness in the regulatory mechanisms of TNF-α synthesis associated with chromatin rearrangement and the synthesis of intermediate signaling molecules, the effect of ASO treatment on the genome manifests later than effects seen after treatment with adalimumab. This was accompanied by a higher level of staining in the micropreparation and the levels of TNF-α in the peripheral blood, which were comparable to those of the control group with RA on the 7th and 14th days, but led to values similar to those of adalimumab by the 21st day.

Several studies have investigated a specific ASO targeting TNF-α used to treat a collagen-induced RA model in mice [42,45]. This substance, analogue of ASO ISIS 104838 directed against human TNF-α [43], showed a strong dose–effect relationship with activity comparable to anti-TNF-α antibodies. The pharmacokinetics of the drug was studied in mice, rats, dogs, and monkeys in the preclinical stage of the study. However, it is impossible to accurately assess the results of the experiment and compare them with ours, because the internal data from the experiments have not been published. However, since in addition to the treatment of RA, monoclonal antibodies have been shown to be effective in treating Crohn’s disease, psoriasis, and other diseases associated with TNF-α overexpression, it is possible to compare the results of ASO testing against TNF-α in a model of chronic colitis. One study, which used a specific ASO against murine TNF-α (ISIS 25302) at doses of 0.25, 2.5, and 12.5 mg/kg injected subcutaneously, reported a dose-dependent decrease in TNF-α mRNA expression in the colon and a decrease in the severity of chronic colitis. A decrease in the severity of chronic colitis was also observed with intravenous administration of the drug at a dose of 1 mg/kg and was demonstrated to be comparable to a similar dose of antibodies against TNF-α (0.03 mg/mouse) [46,47]. It is difficult to compare both the dose of ISIS 25302 used to treat chronic colitis with the dose of Cytos-11 used to treat RA, and the effects of each drug, because they were administered differently and different animal models were used. However, for comparison, in the treatment of colitis, 0.005 to 0.23 mg of the substance was administered via one subcutaneous injection every 2 days for 8 days, with results comparable to the administration of 0.03 mg of an anti-TNF-α antibody. Cytos-11 was administered via subplantar injections using 0.175 mg of the substance twice a week and had a positive effect in the treatment of RA. Again, it is worth noting that the differences in animal models and diseases means that we can only speculate, not confirm, the similarities and differences in the effects of the ASO data. However, as the data from even disparate ASO experiments accumulate, it is important to review them for similarities that could reveal useful trends and patterns.

In another study, researchers investigated a 20-mer-modified ASO with methoxyethyl protection (ISIS 104838), which showed high efficacy, good tolerance, and drug stability during Stage 1 clinical trials. Assessment of its pharmacological effect revealed a dose-dependent, linear, specific decrease in the synthesis of TNF-α by leukocytes in peripheral blood after stimulation with lipopolysaccharide ex vivo; in addition, the maximum concentration of ASO in the plasma proportionally and predictably corresponded to the dose [43]. In our past research with insects, 11-mer ASOs were found to be the most effective and produced optimal effects [44,45,46,47,48,49,50], which is why we used 11-mer phosphorothioate-modified Cytos-11 without methoxyethyl protection. To study its effects, it was administered twice a week for 21 days in a volume of 0.05 mL (3.5 mg/mL) via subplantar injection, followed by evaluation of the effects on the state of the joint and the concentration of TNF-α. This was comparable with the methods used in another study; in one group, ISIS 104838 was injected intravenously at a dose of 0.1, 0.5, 1, 2, 4, or 6 mg/mL on days 1, 8, 10, and 12 of the experiment; in a separate group, it was injected subcutaneously at a concentration of 25, 50, 100, or 200 mg/mL. It was found that ISIS 104838 selectively suppressed TNF-α mRNA expression, reducing its production in keratinocytes and lipopolysaccharide-activated leukocytes. However, the drawback to this study is that the effect of the drug was studied in healthy subjects, so it can only be assumed, not proven, that the drug will have a positive effect on joints affected by RA. In addition, the assessment of TNF-α expression occurred only on the first day (Day 1) and the last day (Day 13) of the experiment. Thus, this work for the first time analyzed the effect of antisense oligonucleotides on the condition of the rheumatic joints.

Comparing these data, we expect that Cytos-11 will have sufficient stability, specificity, and tolerance, as well as linear and dose-dependent drug effects. We plan to confirm this in future studies, as well as searching for possible side effects and non-invasive methods of drug administration. Our experiments showed the effectiveness of the drug in the treatment of RA, demonstrated by the decrease in signs of inflammation, both external and internal edema of the joints, as well as a decrease in lymphocytic infiltration of joint tissues, a decrease in the pannus, and decreases in the levels of TNF-α in the peripheral blood.

## 4. Materials and Methods

### 4.1. Rheumatoid Arthritis Models

Arthritis modeled on human rheumatoid arthritis (RA) was induced by subplantar injection of complete Freund’s adjuvant at a dose of 0.04 mL every third day for 21 days into the left hind paw. After 21 days of injections, the model was allowed to mature for 10 days. An increase in the size of the foot and deformity of the metatarsophalangeal joints served as a signal to begin treatment. The results of the morphological examination showed that by the time the experiment started, the state of the joints corresponded to arthritis with a severity of 1–2 degrees.

### 4.2. Animals and Treatments

A simple, blinded, prospective randomized comparative study was carried out on 30-day-old white Wistar rats. The animals were randomized using the block method [51] and were divided into four groups, each of which had 6 males and 6 females, apart from the control group, where there were 4 more animals. The first group (sham) consisted of non-arthritic animals. The second group (control) consisted of animals treated with 0.9% sodium chloride. Animals in the third group were treated with the drug Humira^®^ at a dose of 0.04 mL once a week via subplantar injection (Humira). Animals in the fourth group were injected with the phosphorothioate antisense oligonucleotide Cytos-11 (sequence 5′-TCC-GTG-CTC-AT-3′), which inhibits the synthesis of TNF-α, at a dose of 0.05 (3.5 mg/mL) ml twice a week for 21 days (Cytos-11). The animals were sequentially withdrawn from the experiment on days 7, 14, and 21 by 4 animals from each group to determine the time of the onset of the therapeutic effect. An additional 4 animals from control group were withdraw on the first day of drug administration.

All manipulations with animals were carried out in accordance with the European Convention for the Protection of Vertebrate Animals Used for Experiments and Other Scientific Purposes and approved by the ethical committee of the Crimean Federal University (Protocol № 10 from 12 November 2020).

### 4.3. Morphology Examination

At the end of treatment, the animals were decapitated under ether anesthesia. A caliper was used to measure the linear dimensions of the thigh, lower leg, foot (foot length, width at the base of the toes, and width at the ankle joint, as well as thickness in the largest dimension). The foot, cleaned of skin, was fixed in formalin for a day and then placed in an acid-free decalcifying solution for 10 days with daily fluid replacement. After that, the feet were dehydrated and soaked in paraffin on an automatic histological processor (Myelstone; Sorisole, Italy). Pieces of foot embedded in paraffin blocks were cut on a Leica RM 2255 microtome (Leica, Nussloch, Germany) into slices with a thickness of 4 μm and stained with hematoxylin and eosin. The preparations were examined and photographed on a DM 2000 microscope (Leica, Wetzlar, Germany) with a digital camera and Plan 5×, Plan 10×, and Plan 40× objectives.

The assessment of the degree of inflammation was carried out according to the scale developed by us using the following criteria:

Leukocyte infiltration rate (1 to 5: 1—single immune cells in the field of view, 2—up to 5% of leukocyte infiltration of the total number of cells in the field of view, 2—6 to 10%, 3—11 to 20%, 4—21 to 50%, 5—more than 50%).

Bone changes (0 to 3: 0—no changes, 1—single changes, 2—pronounced changes, 3—very pronounced changes in the form of tissue structure disturbance, “pitted edge”).

Synovial hyperplasia (0 to 3: 0—absent, 1—weak, 2—medium, 3—strong).

Pannus severity (0 to 5: 0—pannus is absent, 1—small, un-infiltrated, 2—small, infiltrated, 3—medium infiltrated, 4—creeps into the joint cavity, 5—erodes the cartilage).

The degree of destruction of cartilage tissue (0 to 3: 0—no changes, 1—slight changes—fistonic edge, 2—strong changes—about half of the surface is damaged, 3—complete replacement of cartilage with fibrous tissue).

Provisional points were assigned to each animal for each of the criteria. The absence of these signs was assessed as 0 points and the maximum severity of the sign as 3 or 5 points, respectively.

Determination of the thickness of the articular cartilage was carried out using the Image J program with the calibration of the scale using photographs of the reference standard (Stage Micrometer TS-M1 PN 106011) taken with the same camera with similar objectives.

### 4.4. Immunohistochemical Analysis

Immunohistochemistry was used to determine the level of TNF-α in the joint tissues. Paraffin sections were stained with antibodies to TNF-α (Abcam TNFA/1172, clone ab220210, concentration 1:200). Staining was performed in a BondMax semi-automatic immunohistostainer (Leica, Melbourne, Australia). The staining protocol included glass dewaxing, high-temperature unmasking with Bond Epitope Retrieval 2 unmasking solution (pH = 9) for 20 min at 96 °C, peroxidase block, incubation with antibody for 15 min at room temperature, and visualization with the Bond Polymer Refine Detection system.

The staining on the images of the preparations was assessed by scanning them with the Aperio CS2 scanner into the Aperio Image Scope program. Lymphoid cells, synoviocytes, and fibroblasts with distinct membrane, and/or cytoplasmic staining were considered positively stained. Each specimen was assessed in 10 visual fields at three locations: pannus (if any), synovitis, and surrounding soft tissue infiltration. In each case, points were assigned using the following scale:

0—staining of less than 1% of cells or its absence

1—staining of 2–10% of cells

2—staining of 11–30% of cells

3—staining of 31–50% of cells

4—staining of more than 51% of cells.

### 4.5. Linked Immunosorbent Assay

Every 7 days, 1 mL blood was collected from the venous plexus of the floor of the oral cavity into tubes with EDTA. The resulting blood was centrifuged for 5 min at 3000 rpm. The plasma was removed and frozen at −20 °C. After the end of the experiment, the analytes were examined for the content of TNF-α in the peripheral blood using the BMS622 Rat TNF alpha test system on a Multiskan FC plate spectrophotometer (ThermoFisher, Shanghai, China) according to the protocol of the test system manufacturer.

### 4.6. Statistical Analysis

All data obtained as a result of the study were subjected to statistical processing using the STATISTICA 10.0 program. The normality of the trait distribution was assessed using the Kolmogorov–Smirnov method. Using descriptive statistics methods, the median, mean value of the trait, standard deviation, error of the mean, and upper and lower quartiles was obtained. For comparison between groups, the Mann–Whitney test was used. Differences were considered significant if the error probability was *p* < 0.05.

All studies were carried out using equipment at the Center for Collective Use "Molecular Biology" of the Medical Academy named after S. I. Georgievsky (structural unit) and equipment of the Laboratory on Cell Technologies and Elaboration of DNA Medicines of the Taurida Academy (structural unit) of FGAOU VO "Crimean Federal University named after V. I. Vernadsky".

## 5. Conclusions

In this study, ASO Cytos-11 selectively reduced levels of TNF-α in the peripheral blood and cell membranes and reduced joint swelling in rats with rheumatoid arthritis with an efficacy similar to that of adalimumab. By analogy with the results for an ASO used for the treatment of other diseases, Cytos-11 showed a low frequency of immunological reactions, was well tolerated by the rats, and demonstrated the possibility of being used in combination with other drugs. Therefore, it is important to continue studying Cytos-11 to discover any possible undesirable side effects, its potential impact on other markers of inflammation, and the possibility of using it in medical practice.

## Figures and Tables

**Table 1:** Evaluation of the degree of destruction of joint elements in rats in the experiment (Median [1Q;3Q]).

	Days	Infiltration (0–5)	Bone Changes (0–3)	Synovial Hyperplasia (0–3)	Severity of Pannus (0–5)	Destruction of Cartilage Tissue (0–3)	Total Points
Sham		0.0 [0.0;0.0]	0.0 [0.0;0.0]	0.0 [0.0;0.0]	0.0 [0.0;0.0]	0.0 [0.0;0.0]	0.0 [0.0;0.0]
Start of treatment		1.0 [1.0;2.0]	1.0 [0.0;1.0]	1.0 [1.0;1.0]	2.0 [1.0;2.0]	1.0 [1.0;1.0]	6.0 [4.0;7.0]
Control	7	2.0 [1.0;2.0]	1.0 [0.0;1.0]	1.0 [1.0;1.0]	2.0 [1.0;2.0]	1.0 [1.0;1.0]	7.0 [5.0;7.0]
	14	**2.5 [2.0;3.0]**	1.0 [1.0;1.0]	2.0 [2.0;2.0]	3.0 [3.0;3.0]	**2.0 [2.0;2.0]**	**11.0 [10.0;11.0]**
	21	**3.0 [3.0;3.0]**	**1.0 [1.0;1.5]**	**2.5 [2.0;3.0]**	**3.0 [2.5;3.0]**	**2.0 [2.0;2.5]**	**11.5 [11.0;12.5]**
Humira	7	**2.0 [1.0;3.0]**	**1.0 [1.0;1.0]**	**1.0 [1.0;2.0]**	**3.0 [2.0;3.0]**	1.0 [0.0;1.0]	**8.0 [7.0;9.0]**
	14	**2.0 [1.0;3.0]**	**1.0 [1.0;2.0]**	**2.5 [2.0;3.0]**	**3.0 [3.0;3.0]**	1.0 [1.0;1.0]	**9.5 [8.0;11;0]**
	21	**2.0 [1.0;2.0]**	1.0 [0.0;1.0]	**1.0 [1.0;1.0]**	**2.0 [1.0;2.0]**	1.0 [0.;1.0]	**7.0 [5.0;7.0]**
Cytos-11	7	**2.0 [2.0;2.0]**	**1.0 [1.0;2.0]**	**2.0 [2.0;2.0]**	**2.0 [2.0;2.0]**	1.0 [1.0;1.0]	**8.0 [8.0;10.0]**
	14	1.5 [1.0;2.0]	**1.0 [1.0;2.0]**	**2.0 [2.0;3.0]**	**4.5 [3.0;5.0]**	1.0 [0.5;1.5]	**10.0 [8.0;11.0]**
	21	1.0 [1.0;2.0]	1.0 [0.0;1.0]	0.0 [0.0;1.0] *	1.5 [0.0;2.0]	1.0 [1.0;1.0]	5.5 [3.0;7.0]

**Boldface** indicates values that have significant differences from the sham group (*p* < 0.05). * Significant differences in experimental groups vs. the control group.

## Data Availability

Data is contained within the article.
